# Co-Infections by *Fusarium circinatum* and *Phytophthora* spp. on *Pinus radiata*: Complex Phenotypic and Molecular Interactions

**DOI:** 10.3390/plants10101976

**Published:** 2021-09-22

**Authors:** Francesco Aloi, Cristina Zamora-Ballesteros, Jorge Martín-García, Julio J. Diez, Santa Olga Cacciola

**Affiliations:** 1 Department of Agriculture, Food and Environment, University of Catania, Via Santa Sofa 100, 95123 Catania, Italy; francesco.aloi@unipa.it; 2 Department of Agricultural, Food and Forest Sciences, University of Palermo, 90128 Palermo, Italy; 3 Department of Vegetal Production and Forest Resources, University of Valladolid, Av. Madrid 44, 34004 Palencia, Spain; cristinazamoraballesteros@gmail.com (C.Z.-B.); jorgemg@pvs.uva.es (J.M.-G.); juliojavier.diez@uva.es (J.J.D.); 4 Sustainable Forest Management Research Institute, University of Valladolid—INIA, 28040 Madrid, Spain

**Keywords:** pitch canker disease, Monterey pine, *Phytophthora xcambivora*, *P. parvispora*, plant- oomycetes- fungal interactions, gene expression, housekeeping genes, plant-defense molecular mechanisms, PR3, PR5, PAL

## Abstract

This study investigated the complex phenotypic and genetic response of Monterey pine (*Pinus radiata*) seedlings to co-infections by *F. circinatum*, the causal agent of pine pitch canker disease, and the oomycetes *Phytophthora xcambivora* and *P. parvispora*. Monterey pine seedlings were wound-inoculated with each single pathogen and with the combinations *F. circinatum*/*P. xcambivora* and *F. circinatum*/*P. parvispora*. Initially, seedlings inoculated only with *F. circinatum* showed less severe symptoms than seedlings co-inoculated or inoculated only with *P. xcambivora* or *P. parvispora*. However, 30 days post-inoculation (dpi), all inoculated seedlings, including those inoculated only with *F. circinatum*, showed severe symptoms with no significant differences among treatments. The transcriptomic profiles of three genes encoding pathogenesis-related proteins, i.e., chitinase (PR3), thaumatin-like protein (PR5), phenylalanine ammonia-lyase (PAL), and the pyruvate decarboxylase (PDC)-encoding gene were analyzed at various time intervals after inoculation. In seedlings inoculated with single pathogens, *F. circinatum* stimulated the up-regulation of all genes, while between the two oomycetes, only *P. xcambivora* induced significant up-regulations. In seedlings co-inoculated with *F. circinatum* and *P.* *xcambivora* or *P. parvispora* none of the genes showed a significant over-expression 4 dpi. In contrast, at 11 dpi, significant up-regulation was observed for PR5 in the combination *F. circinatum*/*P.* *xcambivora* and PDC in the combination *F. circinatum/P. parvispora*, thus suggesting a possible synergism of multiple infections in triggering this plant defense mechanism.

## 1. Introduction

*Pinus radiata* D. Don, commonly known as Monterey pine, is native to California (Western United States) and has been introduced into areas with a Mediterranean climate of Australia, Chile, New Zealand, South Africa, Spain, and Uruguay [1,2]. Due to the rapid growth and excellent quality of the wood, this pine species, of great interest for forestry, is mainly used for the production of timber [3]. The growth and productivity of *P. radiata* can be severely hampered by various parasites and diseases, resulting in significant economic losses. Several diseases of *P. radiata* have been described; among these, the resinous canker called “pine pitch canker”, caused by the ascomycete *Fusarium circinatum*, is considered one of the most important diseases of *Pinus* species worldwide [4,5].

*Fusarium circinatum* Nelson Nirenberg & O’Donnell, which was formerly named under *Gibberella circinata* Nirenberg & O’Donnell, its teleomorphic stage, is a necrotrophic fungus. *Fusarium circinatum,* listed in the EPPO (European and Mediterranean Plant Protection Organization) quarantine pathogens, has been reported on more than 60 *Pinus* species and on *Pseudotsuga menziesii* (Mirb.) Franco [6,7,8], albeit P. *radiata,* is among the most susceptible ones [5,6,8]. The fungus is able to infect pines of all ages and in all seasons, but mostly during the rainy season [4,5,6,7,8,9]. The first report of this pathogen was on *Pinus virginiana* in the southeastern United States in 1946. Afterward, its presence was reported in other countries, such as Haiti, Japan, South Africa, South Korea, Mexico, Chile, Spain, Italy, Uruguay, Portugal, and Colombia [10,11,12]. The infections originate from lesions that allow the penetration of *F. circinatum* into the susceptible pine organs, including the main stem, secondary branches, and roots [13]. In nurseries, the main symptom of the pine pitch canker consists of dying off of the seedlings (damping off), while in plantations, the most common symptoms are the drying of the branches and cankers on the stems, with the production of abundant resinous exudate, which leads to the death of the apex of the plant, resulting in stunted growth and reduced crown and stem volume as a consequence of this malformation [4,10,14].

*Phytophthora* spp. are a group of major pathogens of *Pinus* species. The genus *Phytophthora* (Oomycetes, Stramenopila) encompasses at least 200 recognized species, considering they were over 180 by 2018, and most of these species are plant pathogens [15,16,17,18,19,20,21,22,23,24,25]. The majority of *Phytophthora* spp. are soil-borne and prevalently infect the roots and the basal stem. However, many soil-borne *Phytophthora* species have adapted, at least temporarily, to an aerial lifestyle and are able to infect leaves, twigs, and fruit [18]. Stem cankers and root and crown rot are the most common symptoms induced by these pathogens on woody hosts, both in nurseries and on mature trees [18,26,27,28,29]. Several *Phytophthora* species were reported to be associated with damping-off of seedlings, stem cankers, root rot, and decline of *Pinus* species, including *P. austrocedri*, *P. cactorum*, *P. xcambivora*, P. *capsici, P. castaneae*, *P. cinnamomi sensu lato*, *P. citricola sensu lato*, *P. citrophthora*, *P. cryptogea*, *P. crassamura, P. drechsleri, P. fallax, P. humicola, P. kernoviae, P. nicotianae, P. obscura, P. pini, P. plurivora*, *P. syringae,* and other unidentified *Phytophthora* spp. [29,30,31,32,33,34,35]. *Pinus* plants affected by *Phytophthora* crown and root rot show growth reduction, chlorosis, and decline [18]. The genus *Phytophthora* includes species that have a significant impact in European pine forests and are believed to be among major pine pathogens occurring together with *F. circinatum*. Infections by these Oomycetes are favored by climatic factors, such as high humidity, rain, and warm temperatures, which are known to be conducive also for infections by *Fusarium* spp. Therefore, interaction, including co-infection and synergy, between species of these two genera of pathogens can be assumed to be very probable [34].

During pathogen attack, different metabolic pathways are activated in the plant, including changes in primary energy-producing metabolism, plant defense mechanisms, and hormone signaling. Defense mechanisms are controlled by a series of genes, individually or synergistically involved in the resistance traits of plants [35,36,37,38]. As in the majority of plants, in conifers, the hypersensitive responses are the most commonly induced defense mechanisms against pathogens. These mechanisms include early lignification of fibers and the production of terpenoids and alkaloids that are phenolic compounds [39]. These responses are activated upon recognition of a pathogen by triggering signal transduction mechanisms that stimulate secondary metabolic pathways resulting in the production of defense compounds, which represents the result of the expression of a diverse range of genes [36,37,38,39,40]. Previous studies have identified a number of genes implicated in response to pathogen infection in coniferous species [41,42]. These encompass genes codifying for pathogenesis-inherent proteins (PR proteins), which represent the most numerous group of plant defense proteins, as well as genes coding for secondary metabolites with a wide range of antifungal activity, like the ones linked to the phenylalanine metabolic pathway [43,44]. Phenylalanine is pivotal in connecting primary and secondary plant metabolism and is being utilized by phenylalanine ammonia-lyase (PAL) for the synthesis of a number of compounds that are critical to the plant under stress situations [45]. In addition to PAL, other major pathogenesis-related (PR) proteins, including PR3 (chitinase) and PR5 (thaumatin-like protein), are commonly involved in resistance to fungal pathogens [46]. Also, plant respiration is usually enhanced after infection of the pathogen. Pyruvate decarboxylase (PDC) is triggered under stressed circumstances and can transform pyruvate into acetaldehyde and CO2 under aerobic fermentation. Acetaldehyde may be converted to ethanol during fermentation or be transformed into acetyl-CoA by pyruvate dehydrogenase and enter the tricarboxylic acid (TCA) cycle [47]. While the expression levels of genes encoding for pathogenesis-related proteins and PDC [46] has been extensively investigated in pathosystems where a single pathogen is involved, little information is available on the relative expression levels of genes that respond to the plant-pathogen interaction in more complex pathosystems where two or more pathogens simultaneously infect the host-plant.

The aim of this study was to investigate: i. the phenotypic response of Monterey pine to co-infections by *F. circinatum* and *Phytophthora* species, and ii. the relative expression levels of genes encoding for pathogenesis-related proteins and PDC in seedlings of Monterey pines co-infected by these pathogens.

## 2. Results

### 2.1. Symptom Progression

**Figure 1:**
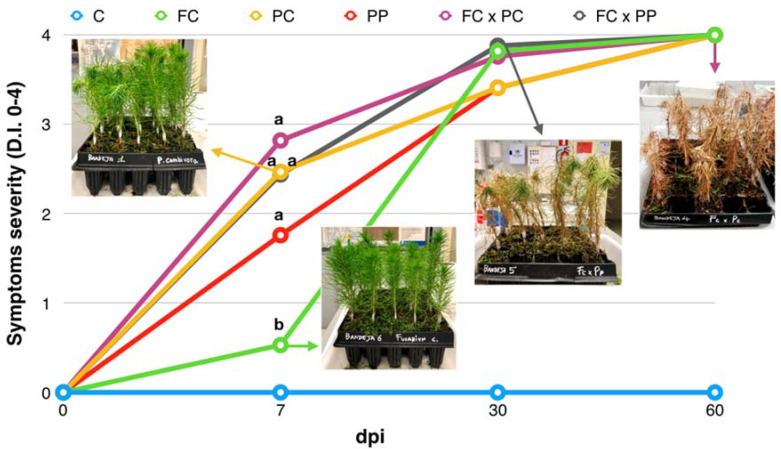
Time course of symptoms progression in *Pinus radiata* seedlings non-inoculated (C) or inoculated with *Fusarium circinatum* (FC), *P. xcambivora* (PC), *P. parvispora* (PP), *F. circinatum* ∗ *P. xcambivora* (FC × PC) and *F. circinatum* ∗ *P. parvispora* (FC × PP). Symptom severity was expressed as mean value of disease index (D.I.) at 7, 30, and 60 days post-inoculation (dpi). Images show the type of symptoms at each time interval post-inoculation. At each time interval, values sharing the same letters are not significantly different according to Tukey’s honestly significant difference (HSD) test (*p* ≤ 0.05).

A common symptom on inoculated seedlings was the folding of the apex (damping-off). Other symptoms included restricted resin exudations and necrotic lesions around the wound, which were visible in the advanced stage of the disease. Non-inoculated control seedlings were asymptomatic. In detail, 7 days post-inoculation (dpi), the first symptom on all inoculated seedlings was the damping-off of the apex; all inoculated seedlings showed severe symptoms with disease severity in terms of disease index (D.I.) ranging from 2 to 3, except seedlings inoculated only with *F. circinatum* (FC) which initially showed significantly less severe symptoms (mean D.I. 0.5) (Figure 1). Thirty dpi, seedlings from all the treatments, including those inoculated only with *F. circinatum*, showed an advanced state of decay (D.I. ranging from 3 to 4). At this evaluation date, additional symptoms were wilting and browning of needles, indicating the plant was about to die. As a matter of fact, the death of all inoculated seedlings occurred 60 dpi.

**Figure 2:**
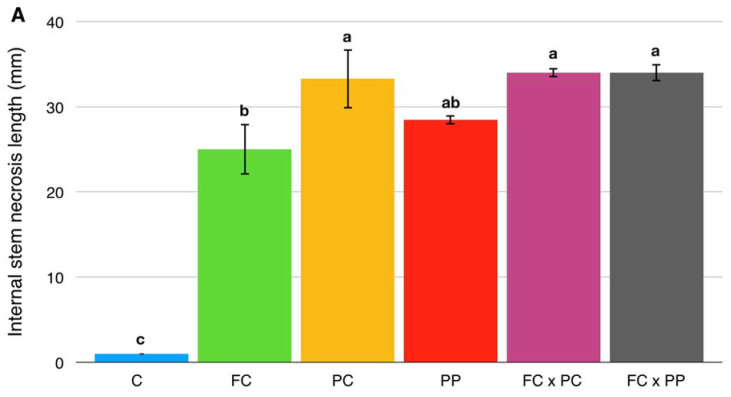

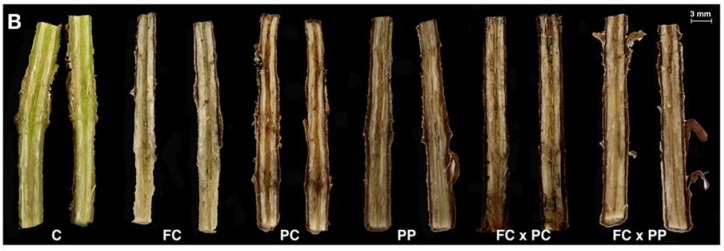
(**A**) Mean values of the length of internal stem necrosis (mm) in *Pinus radiata* seedlings non-inoculated (C) or inoculated with *Fusarium circinatum* (FC), *P. xcambivora* (PC), *P. parvispora* (PP), *F. circinatum* ∗ *P. xcambivora* (FC × PC) and *F. circinatum* ∗ *P. parvispora* (FC × PP), 14 days post-inoculation (dpi), when more than 50% of the inoculated seedlings from each treatment showed disease symptoms. Data are presented as mean ± SE of four biological replicates per treatment. Values sharing the same letters are not statistically different according to Tukey’s honestly significant difference (HSD) test (*p* ≤ 0.05). (**B**) Stem internal necrosis from representative samples of *Pinus radiata* seedlings non-inoculated (C) or inoculated with *Fusarium circinatum* (FC), *P. xcambivora* (PC), *P. parvispora* (PP), *F. circinatum* ∗ *P. xcambivora* (FC × PC) and *F. circinatum* ∗ *P. parvispora* (FC × PP) observed using a zoom stereomicroscope when more than 50% of the inoculated seedlings of each treatment showed disease symptoms (14 dpi).

The time course of symptom progression is summarized in Figure 1. All inoculated seedlings, with no exceptions, showed an extended necrotic stem lesion, while non-inoculated control seedlings did not show any symptoms (Figure 1 and Figure 2B). The length of the internal stem lesions was measured at 14 dpi, when more than 50% of the inoculated seedlings in each treatment showed symptoms. The statistically significant differences between the length of stem lesions in seedlings inoculated with single pathogens or their combinations (Figure 1 and Figure 2A) were consistent with the severity of visual symptoms, as evaluated 7 dpi (Figure 1).

### 2.2. Housekeeping Gene Selection

In order to obtain reliable results, the expression level of target genes was standardized using internal control genes, also referred to as reference or housekeeping genes [47,48]. The expression level of these genes should be quite stable between diverse samples and environmental conditions. Until now, there have been no housekeeping genes that have been validated for the purpose of gene expression on conifers being infected by *F. circinatum* or similar necrotrophic fungi [46]. The stability of the three selected housekeeping genes was estimated by using the comparative delta Ct method and geNorm software. For both methods, ubiquitin (UBQ) was the more stable housekeeping gene, while actin (ACT) and β-Tubulin (TUB) TUB had the highest delta Ct values and the highest M values when analyzed with the geNorm software. The current study indicated that UBQ was the overall most stable reference gene for P. radiata that was infected with F. circinatum or similar necrotrophic fungi. This evidence was congruent with the results of other research aiming to provide validation of reference genes on plants under biotic and abiotic stress situations [46,49,50,51]. Therefore, UBQ was used for normalizing qRT-PCR results.

### 2.3. Differential Expression of Candidate Genes

**Figure 3:**
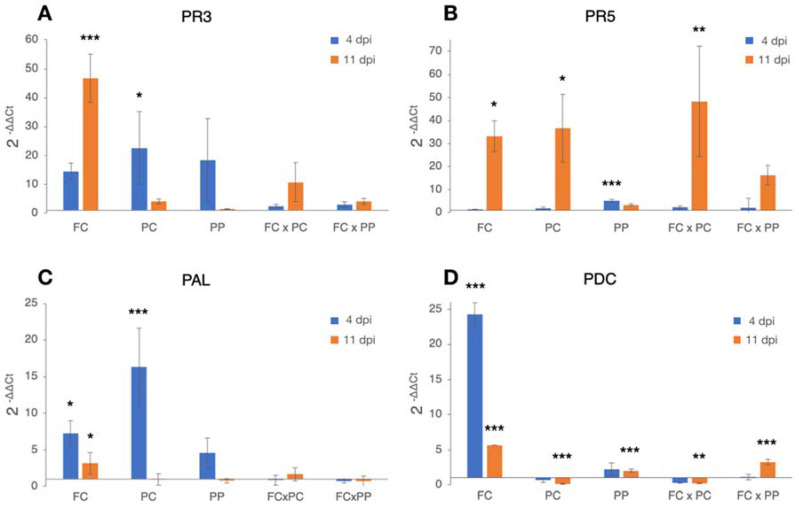
Differences in the expression levels of PR3- (**A**), PR5- (**B**), PAL- (**C**) and PDC- (**D**) -encoding genes in *Pinus radiata* seedlings at 4 (blue bars) and 11 (orange bars) days-post-inoculation (dpi) with *Fusarium circinatum* (FC), *P. xcambivora* (PC), *P. parvispora* (PP), *F. circinatum* ∗ *P. xcambivora* (FC × PC) and *F. circinatum* ∗ *P. parvispora* (FC × PP). Columns with asterisks are statistically different according to Dunnett’s test (* *p* < 0.05, ** *p* < 0.01, *** *p* < 0.001), compared with their calibrator (i.e., wounded and non-inoculated control seedlings).

The analysis of the transcriptomic profile of the candidate genes (PR3, PR5, PAL, and PDC) evidenced variable responses depending on both the treatment and the time interval after inoculation. The PR3-encoding gene for chitinase resulted up-regulated in all treatments at both time intervals after inoculation (4 and 11 dpi), with significant differences over the non-inoculated control in seedlings inoculated with *P. xcambivora* (PC) (4 dpi) and *F. circinatum* (FC) (11 dpi) (Figure 3A).

The PR5-encoding gene for thaumatin-like protein, like the PR3, had a generalized up-regulation pattern, although it was significantly up-regulated mainly at 11 dpi. In particular, at 4 dpi, a weak significant up-regulation was observed only in seedlings inoculated with *P. parvispora*, while 11 dpi a marked up-regulation was observed in *F. circinatum-* (FC) and *P. xcambivora-*(PC) inoculated seedlings as well as in seedlings inoculated with *F. circinatum* ∗ *P. xcambivora* (FC × PC), and *F. circinatum* ∗ *P. parvispora* (FC × PC) (Figure 3B).

The PAL-encoding gene resulted generally up-regulated 4 dpi only in treatments that received the tested pathogens (FC, PC, PP) singularly, although the up-regulation was significant only in seedlings inoculated with *F. circinatum* (FC) and *P. xcambivora* (PC). *Fusarium circinatum* (FC) was the only significantly up-regulated treatment at 11 dpi (Figure 3C).

Finally, the PDC-encoding gene was significantly modulated in all the treatments, although with opposite trends. The strongest up-regulation was observed in seedlings from treatments with *F. circinatum* (FC) only at both time intervals (4 and 11 dpi). The gene was also up-regulated at both time intervals in the treatment with *P. parvispora* (PP), although the modulation was significant only at 11 dpi. Generalized significant down-regulation was recorded in seedlings from treatments with *P. xcambivora* alone (PC) and with *F. circinatum* (FC × PC). Finally, in the interaction system *F. circinatum* ∗ *P. parvispora* (FC × PP), the gene was significantly up-regulated only at 11 dpi (Figure 3D).

## 3. Discussion

The complex interactions of plant-multiple microorganisms represent a major topic of modern Plant Pathology [52,53]. Indeed, several studies started to investigate the mechanisms by which microorganisms interact with each other and affect the phenotypic and genetic response of plants as a consequence of their pathogenetic activity [52,53,54,55]. In order to try to elucidate how simultaneous infections by different plant pathogens can affect the progression of a specific plant disease traditionally imputed to a single pathogen, this study addressed the plant-microorganism interaction in the pine pitch canker pathosystem (*Pinus radiata*-*Fusarium circinatum*) under the additional influence of two aggressive *Phytophthora* species that cause stem cankers of pines. The synergism between pathogens is key to understanding the etiology and pathogenesis mechanisms of several complex diseases. Examples of this type of disease involving a *Phytophthora* species include the *Phytophthora*-*Diaprepes* complex and the Huanglongbing syndrome of citrus [56]. However, special attention was paid to the interactions between pathogenic fungi, including *Fusarium* species [57,58]. A typical example of a complex fungal disease is the esca disease of grapevine, which is present across many regions worldwide and is caused by diverse fungal pathogens present alone or in combination, including *Ilyonectria* spp., *Phaeomoniella chlamydospora*, *Togninia* spp., and Botryosphaeriaceae species [59,60]. A more recent study demonstrated that co-infection of several species of Botryosphaeriaceae and *Ilyonectria* results in a more severe decline of young grafted grapevines in the field [60]. Similarly, laboratory experiments further confirmed that co-inoculation of *Ilyonectria* and Botryosphaeriaceae isolates induced an increased disease severity compared to single inoculation of *Ilyonectria* isolates [61].

In the present study, the experimental approach was aimed at investigating the phenotypic and gene-mediated response of *P. radiata* plants to an infective process due to the simultaneous infection by two plant pathogens, *F. circinatum* and *P. xcambivora* or *F. circinatum* and *P. parvispora*. Previous studies reported synergic effects of diverse pathogens in the phenotypic plant response to complex diseases involving *Fusarium* species and other fungus and oomycete pathogens belonging to different genera. In the case of cassava root rot, the pathogens involved were *Fusarium* sp., *Botryodiplodia theobromae,* and *Armillaria* sp. [61]. In the gray necrosis of hazelnut, the species of fungal pathogens involved were *Alternaria* sp., *Fusarium* *lateritium,* and *Phomopsis* sp. [62]. *Pythium* sp., *Fusarium* sp., *Cylindrocarpon* sp., and *Rhizoctonia* sp. were responsible for the root rot of strawberry [63], and *Pythium* sp., *Rhizoctonia* sp., and *Fusarium* sp. were responsible for the root disease of *Trifolium vesiculosum* [64]. Potential interactions between *F. circinatum* and other fungal pathogens can also be expected. Indeed, the genera *Heterobasidion*, *Armillaria*, and *Phytophthora* include root and butt rot pathogens with a wide distribution and high impact in pine forests in Europe, and their infections are favored by factors such as high humidity, which are also favorable to *Fusarium* spp. [65,66]. Therefore, although a co-occurrence of *F. circinatum* has been only reported so far for *Diplodia sapinea* [66,67], it is most likely that diverse aggressive fungal species can infect simultaneously with *F. circinatum*. In the present study, the possible interaction of *F. circinatum* and aggressive *Phytophthora* species was investigated for the first time. Results showed the simultaneous infection by these pathogens exacerbated the severity of symptoms caused by *F. circinatum* alone in the early stages of the infection process, possibly due to more rapid colonization of the stem of pine seedlings by *Phytophthora* spp. In this respect, the co-infection has produced an additive rather than a synergistic effect. However, it has to be considered that, in this case, it would be difficult to evaluate any synergistic effect only on the basis of phenotypic response as all three tested pathogens alone had lethal effects on pine seedlings.

The study of gene expression was expected to be more effective than symptom severity to get a better insight into the response of the plant to multiple infections. It is well known that in plants, the infection by a pathogen triggers the activation of the main resistance mechanism, the systemic acquired resistance (SAR), which provides long-term resistance throughout the plant to subsequent infection by different pathogens [51]. This kind of resistance is correlated with the synthesis of pathogenesis-related (PR) proteins, which, in turn, is mediated by the up-regulation of genes encoding enzymes involved in the biosynthesis of salicylic acid (SA) [68]. Plant cellular respiration is also usually stimulated by pathogenic infections [45,50,51,52]. In such a kind of oxygen stress condition, it has been observed that plants respond by the synthesis of pyruvate decarboxylase (PDC), which then converts the pyruvate into acetaldehyde and CO_2_ and makes possible the production of energy [46]. In order to decrypt how the simultaneous infection by *F. circinatum* and *Phytophthora* species affects the plant defense response, this study describes the modulation of the SAR and plant cellular respiration by the analysis of the transcriptomic profile of three genes encoding for pathogenesis-related proteins (PR3, PR5, and PAL) as well as that of the pyruvate decarboxylase- (PDC) encoding gene.

The results from the interaction systems (*F. circinatum* ∗ *P. xcambivora,* and *F. circinatum* ∗ *P. parvispora*) showed that, at the early stages of the infection (4 dpi), none of the studied encoding genes had a significant modulation over the non-inoculated while at 11 dpi, significant up-regulation was exclusively reported for PR5 in the treatment *F. circinatum* ∗ *P.* *xcambivora* and PDC in *F. circinatum* ∗ *P. parvispora*. Interestingly, a significantly higher up-regulation of gene coding for PR5 was observed in the *F. circinatum* ∗ *P.* *xcambivora* interaction, suggesting a possible synergism of multiple infections in triggering this plant defense mechanism. Results from inoculations with a single pathogen overall evidenced that *F. circinatum* markedly stimulated the up-regulation of all the studied genes mainly at the late stages of infection, while between the two *Phytophthora* species, only *P.* *xcambivora* seemed to stimulate significant up-regulations. These effects can be explained assuming that: i. the competition between pathogens for the infection site and substrate could have determined a delay in the onset of the infective process by *F. circinatum* and consequently of the plant defensive response, and ii. multiple infections might have repressed the expression of defense-related genes, with the only exception of the PR5 encoding gene. In this respect, co-infection by aggressive pathogens might act synergistically by circumventing or silencing plant defense responses, thus exacerbating the final effect of the infection. Considering that this is the first study that investigated the pine response to multiple infections by different pathogens simultaneously, further studies are needed to validate these two hypotheses.

## 4. Materials and Methods

### 4.1. Plant Material

Ten-month-old *Pinus radiata* seedlings (Spanish provenance) were used for the experiment. Seedlings had an average height of 19.84 ± 1.8 cm and an average diameter at the soil level of 0.34 ± 0.03 cm. They were maintained in a greenhouse at 20–22 °C and a photoperiod of 16 h light/8 h darkness and inoculated after two weeks of acclimation.

### 4.2. Fungal Inoculum and Inoculation Methods

An isolate of *F. circinatum* from *Pinus radiata* (Fc 072) sourced in Spain and two isolates of *Phytophthora*, *P.* *xcambivora* from *Quercus ilex* (PH 14.012) sourced from forest soil in Spain, and *P. parvispora* recovered from rhizosphere soil of *Salix pedicellata* in a riparian forest in Sicily [19] were used for plant inoculation. Spore suspensions (*F. circinatum* microconidia and *Phytophthora* zoospores) were used for inoculum.

*Fusarium circinatum* was grown on potato dextrose agar (PDA) at 25 °C in the dark until the mycelium covered at least 90% of the Petri dish. Three to five pieces of mycelium (5 mm diameter) were grown under agitation on potato dextrose broth (PDB) for at least 24 h. Microconidia were then recovered by centrifugation, rinsed, and resuspended in sterile distilled water (s.d.w.). The final conidium concentration was adjusted to 10^6^ conidia/mL using a hemocytometer.

*Phytophthoraxcambivora* and *P. parvispora* were first grown on V8 juice agar (V8A) at 20° C in the dark for 7 days. For the zoospore production, mycelium plugs from these colonies were flooded in s.d.w. with the addition of autoclaved soil extract and incubated for 2–3 days at 20–22 °C with a 16/8 h photoperiod. Sporangia formation was monitored during this incubation period, and once mature sporangia were observed, the plates were cold shocked by incubation at 4 °C for 45 min after which they were removed and left at room temperature for 1 h to stimulate zoospore release. The zoospores were removed from the plates, pooled together, and used for the inoculation. The zoospore concentration was determined by using a hemocytometer and standardized to 10^6^ zoospores/mL.

The experimental scheme consisted of the following treatments: i. wounded plants control (C); ii. plants inoculated with *F. circinatum* (FC); iii. plants inoculated with *P.* *xcambivora* (PC); iv. plants inoculated with *P. parvispora* (PP); v. plants inoculated with *F. circinatum* ∗ *P. xcambivora* (FC × PC); vi. plants inoculated with *F. circinatum* ∗ *P. parvispora* (FC × PP). For the inoculation, 180 seedlings of *Pinus radiata* were used, 30 replicates per treatment (i.e., 1 forest tray of 30 cells). The stem surface of plants from all treatments was wounded by two cuts at the same height, at two opposite sides of the stem, using a sterile scalpel; 10^6^ spores (*F. circinatum* microconidia and *Phytophthora* zoospores) were then applied at one of the two wounds; the other wound received sterile distilled water in plants from treatments ii., iii. and iv., while each wound received singularly one of the two pathogens in plants from treatments v. and vi. Wounds were then sealed with Parafilm^®^ to prevent desiccation and contamination. Control plants were equally wounded and received an equal volume of s.d.w. After inoculation, controls and inoculated plants were kept in separate climate chambers under controlled conditions at a temperature of 20 ± 2 °C, 40–50% relative humidity, a photoperiod of 16/8 h light/dark for 60 days and irrigated 30 min per day.

### 4.3. Evaluation of Symptoms and Internal Necrosis Length

The typical symptoms of pine pitch canker disease are tip dieback, wilting, browning of needles, and resin formation and were evaluated and monitored once a week. The seedlings were visually scored for disease symptoms according to a 0–4 Disease Index (D.I.) empirical scale proposed by Correll et al. [69]: 0 = healthy plant or no symptoms; 1 = resin and/or necrosis at the point of inoculation and healthy foliage; 2 = resin and/or necrosis beyond the point of inoculation; 3 = severe wilting and noticeable dieback or damping-off and 4 = dead plant. Symptoms were evaluated at 3-time intervals after inoculation, i.e., 7, 30, and 60 days post-inoculation (dpi). To re-isolate the pathogens, stem cuttings were plated onto PDA or V8A and incubated at 20–22 °C for 7 days. The internal stem lesion length (mm) was also measured in 4 cm longitudinal stem cuts of three biological replicates per treatment when more than 50% of the inoculated plants showed disease symptoms (14 dpi). Images of the necrosis were obtained using a zoom stereomicroscope.

Data were analyzed by using one-way ANOVA followed by Tukey’s HSD test (Honestly Significant Difference) as a post hoc test (R software). Differences at *p* ≤ 0.05 were considered significant.

### 4.4. Sample Collection, RNA Extraction, and cDNA Synthesis

Stem fragments of approximately 4 cm in length, 1 cm above the inoculation point, were sampled for RNA extraction at 4 and 11 dpi, using 5 seedlings per treatment at each sampling time interval. Samples were stored at −80 °C until used. Total RNA was extracted by using Spectrum™ Plant Total RNA Kit (Sigma-Aldrich, St. Louis, MO, USA) and quantified using a Qubit 4 Fluorometer (Invitrogen, Waltham, MA, USA). The integrity of RNA samples was visualized by loading 5 μL of RNA on a 1.5% agarose gel stained with SYBR Safe. cDNA was synthesized using Revert Aid Reverse Transcriptase (Thermo Fisher Scientific, Waltham, MA, USA), random hexamers, and 1μg of RNA in 20 µL reactions following the manufacturer’s specifications.

### 4.5. Selection of Primers and Housekeeping Genes

The primers used to amplify the four candidate genes, namely, PR3, PR5, PAL, and PDC [33,34,35,36,37,38,39], and three housekeeping genes, namely actin (ACT), β-tubulin (TUB), and ubiquitin (UBQ), are shown in Table 1. Primers were selected on the basis of previous studies [45,46,47,48,49,50]. Housekeeping genes’ stability was analyzed using the geNORM v. 3.4 software and the comparative delta-Ct method [70,71,72]. For the latter, the reference gene analysis tool refFinder (http://www.leonxie.com/referencegene.php) was used. After the analysis, the most stable reference gene was used for the normalization of the gene expression data.

### 4.6. Relative Expression of Candidate Genes

Amplifications were performed by using the iCycler iQ™ Real-Time PCR Detection System (Biorad). Reactions were performed in a total volume of 20 μL by mixing 10 ng of cDNA with 1 μL of 10 μM of each primer and 10 μL of PowerUp™ SYBR™ Green Master Mix (2X) (Applied Biosystems). qRT-PCR experiments were carried out in triplicate. The thermo-cycling conditions were 2 min at 50 °C (UDG activation), 2 min at 95 °C (Dual-Lock™ DNA polymerase) followed by 40 cycles of two steps: 95 °C for 15 s (denaturation) and 65 °C (annealing/extension) for 1 min. The quantification of gene expression was carried out by using the 2^−^^ΔΔCt^ method where ΔΔCt = (Ct of target gene-Ct of reference gene) sample-(Ct of target gene-Ct of reference gene) calibrator and Ct is the threshold cycle of each transcript, defined as the point at which the amount of amplified target reaches a fixed threshold above the background fluorescence and using housekeeping genes for data normalization as described by Vandesompele et al. [70,71].

Data on gene expression were analyzed by using one-way ANOVA followed by Dunnett’s multiple comparisons test by using R software. Differences at *p* ≤ 0.05 were considered significant.

## 5. Conclusions

This study investigated for the first time the effects of co-infections of pine seedlings by diverse aggressive pathogens, the true fungus *Fusarium circinatum,* causing pine pitch canker disease, and the oomycetes *Phytophthora xcambivora* and *P. parvispora,* causing crown and root rot. According to the aim, it provided a preliminary contribution to the understanding of the genetics of plant defense mechanisms in multiple infections. Based on the development of symptoms and the genetic analysis of the transcriptomic profile of the pyruvate decarboxylase-(PDC)-encoding gene and three genes encoding pathogenesis-related proteins (PR3, PR5, and PAL), two tentative hypotheses were proposed: i. the competition between pathogens could have delayed the infective process by *F. circinatum* and the plant defense response; ii. co-infection might have repressed the expression of defense-related genes, thus exacerbating the severity of the disease.

## Figures and Tables

**Table 1:** Primer sequences used for the amplification of housekeeping and candidate genes studied in the multi-factorial system *Pinus radiata*-*Fusarium circinatum*-*Phytophthora* species.

Gene	Primer Sequence	GenBank ID	Functions/Putative Functions	References
Chitinase (PR3)	F: TGGCAACACGGACGCCCATT	HM219849.1	Hydrolyzation of chitin.	[45,50,73,74]
	R: ACCGGCGTCGTTTCTGTGCTT			
Thaumatin-like protein (PR5)	F: AGGAGCGCGTGTGATGCGTT	JQ015859.1	Involved in cell wall damage and formation of pores on the plasma membrane.	[45,46,47,48,49,50,75,76]
	R: TGAAAGTGCTGGTGGCGTCGT			
Phenylalanine ammonia-lyase (PAL)	F: TGCTGGCCACTGTGAAGCAGA	AY641535.1	Lignin and phenolic accumulation in plants. Cinnamic acid synthesis	[45,46,47,48,49,50,77,78]
	R: TCGCAGAAACGGCCTGGCAA			
Pyruvate decarboxylase (PDC)	F: CCCGCAAACAATGACGTGGGGT	JQ264496.1	Involved in aerobic fermentation.	[45,46,47,48,49,50,79,80]
	R: TGCGAGCAGATGGTCCAGCA			
Actin (ACT)	F: TGGACCTTGCTGGGCGTGATCT	GQ339779.1	Major component of cytoskeleton microfilaments.	[45,46,47,48,49,50,81]
	R: ACAATCTCGCGCTCTGCGGT			
β-Tubulin (TUB)	F: AAGGGGGTCAGTGTGGCAACCA	KM496536.1	Structural units of the cytoskeleton microtubes	[45,46,47,48,49,50,82]
	R: ACAGCCCGCGGAACAAACCT			
Ubiquitin (UBQ)	F: AGCCCTTATGCCGGAGGGGTTT	AF461687.1	Participates in protein recognition by the proteasome	[45,47,50]
	R: AGTGCGGGACTCCACTGTTCCT			

## Data Availability

Row Data can be shared upon a reasonable request.
